# MAPT rs242562 and GSK3B rs334558 are associated with Parkinson’s Disease in central China

**DOI:** 10.1186/1471-2202-15-54

**Published:** 2014-04-29

**Authors:** Lan Yu, Jinsha Huang, Desheng Zhai, Ling Liu, Kexin Guo, Xi Long, Jing Xiong, Zhentao Zhang, Youpei Wang, Ying Zhao, Ping Wu, Dingan Wang, Zhicheng Lin, Jing Wu, Nian Xiong, Tao Wang

**Affiliations:** 1 Department of Neurology, Union Hospital, Tongji Medical College, Huazhong University of Science and Technology, 1277 Jiefang Road, Wuhan 430022, Hubei, China; 2 Department of Radiology, Union Hospital, Tongji Medical College, Huazhong University of Science and Technology, Hubei 430022, China; 3 Department of Neurology, Renmin Hospital of Wuhan University, Wuhan 430060, China; 4 Department of Public Health, Xinxiang Medical University, Xinxiang 453003, China; 5 School of Pharmacy, Xinxiang Medical University, Henan 453003, China; 6 Hefeng Central Hospital, Hefeng, Enshi, Hubei 445800, China; 7 Department of Psychiatry, Harvard Medical School, Boston, MA 02114, USA; 8 Laboratory of Psychiatric Neurogenomics, Division of Alcohol and Drug Abuse, and Mailman Neuroscience Research Center, McLean Hospital, Belmont, MA 02478, USA; 9 Harvard NeuroDiscovery Center, Boston, MA 02114, USA; 10 Key Laboratory of Environment and Health, Ministry of Education & Department of Epidemiology and Biostatistics, School of Public Health, Tongji Medical College, Huazhong University of Science and Technology, Hubei 430030, China

**Keywords:** *MAPT* H1 haplotype, *GSK3β*, Parkinson’s disease, Genetic risk factor

## Abstract

**Background:**

Microtubule-associated protein tau (MAPT) is a neuronal protein involved in the pathogenesis of several neurodegenerative diseases including Parkinson’s Disease (PD). Glycogen synthase kinase 3 beta (GSK3B) catalyzes phosphorylation in multiple sites of tau protein. However, whether or not there is any association between the *GSK3B* gene alteration, *MAPT* haplotype and PD remains unexplored in Chinese population, especially in central Chinese population.

**Results:**

Here, we aimed at studying the effect of *MAPT* rs242562 and *GSK3B* rs334558 on the risk of PD by performing a case-control association study in central China. Our data showed that all PD patients and controls were of *MAPT* H1/H1 diplotype in our study, thus confirming that the distribution of the *MAPT* H1 haplotype is common in China. GG genotype of *MAPT* rs242562 serves protection effect on PD risk in central Chinese population, while genotype of *GSK3B* rs334558 showed no difference between PD patients and controls.

**Conclusions:**

We conclude that the *MAPT* rs242562 is associated with PD in central China in the background of *MAPT* H1/H1 diplotype. The GG genotype of rs242562 displays protection against PD in subgroup with *GSK3B* rs334558 T carrier.

## Background

Parkinson’s disease (PD) is a common neurodegenerative disease in elders, characterized by the loss of dopaminergic neurons in substantial nigra and the formation of Lewy bodies [1]. PD affects approximately 1% of population aging over 60 years. Disease onset before 50 years of age defines as early-onset Parkinson's disease (EOPD) while that over 50 years of age, as late onset Parkinson’s disease (LOPD). The etiology and pathogenesis of PD, mainly considered to be related with aging, genetic and environmental factors, is incompletely understood. Recently, linkage analysis and genomewide association studies have shown that *PARK1-PARK18*, *POLG*, *Glycogen Synthase kinase-3B (GSK3B)*, *Microtubule-associated protein tau (MAPT)* and other genes are associated with PD risk.

The *MAPT* gene is localized in a region of extended linkage disequilibrium (LD) on chromosome 17q21, containing 16 exons and encoding microtubule-associated proteins tau. Tau proteins, constituting a family of six isoforms ranging between 352-441 amino acids, are widely expressed in the nervous system. Excessive phosphorylation of tau proteins leads to abnormal intracellular aggregation, formatting of the double helix fiber filament, producing the nerve fiber tangles, and finally neurodegeneration [2]. *MAPT* gene, defines two extended haplotype, H1 and H2. The determination of MAPT haplotype H1/H2 is attributed to the presence of a 238 bp deletion between exons 9 and 10 of MAPT H2 haplotype [3]. H1 haplotype is reportedly related to PD susceptibility [4]. Concerning sub-haplotypes, there is a positive correlation between *MAPT* H1 haplotype and PD, including SNP rs242562 (A/G) and rs2435207 (G/A). The A-A sub-haplotype is associated with PD patients in a Norwegian population, while the G-A sub-haplotype for these two SNPs is associated with PD subjects in Greece [5-7]. However, at present, little is known about the correlation between *MAPT* gene polymorphism and PD patients in China, especially in central China area. Central China includes six widely agricultural provinces namely Hunan, Hubei, Jiangxi, Henan, Shanxi and Anhui.

GSK3B protein is widely expressed in all tissues, with particularly abundant levels in the brain [8]. The fundamental role of GSK3B in intracellular neuronal signaling systems is underpinned by its ability to phosphorylate several proteins that contribute to the structural characteristics and dynamics of neuronal cells. In addition, it has been proven that GSK3B catalyzes phosphorylation in multiple sites of tau protein [9], and it is an important pathogenic protein kinase for PD. Previous data indicate that the genetic alteration of *GSK3B* and its interaction with *MAPT* haplotypes are collectively related to PD morbidity rate in a Greek cohort [3,10]. However, the association between the *GSK3B* gene alteration, *MAPT* haplotype and PD has not been previously explored in Chinese population.

In this study, our database contains 211 PD patients and 279 controls from central China. Our study examined the possible association between *GSK3B* promoter single nucleotide polymorphism (SNP) rs334558, *MAPT* haplotype, *MAPT* intron SNP rs242562 and PD in central China.

## Methods

### Subjects

We recruited 211 unrelated sporadic PD patients (average age at diagnosis: 57.23 ± 12.9 years; 137 males, 74 females) diagnosed according to the criteria of the UK Parkinson's Disease Society Brain Bank in Union hospital, Tongji Medical College, Huazhong University of Science and Technology (TJMC & HUST). The control group consisted of 279 individuals (average age: 53.72 ± 13.32 years; 178 males, 101 females). This study was approved by the Ethical Committee of TJMC & HUST. The ethics and written informed consent was obtained from all subjects.

### Genotyping

Genomic DNA was extracted from venous blood using standard methods. Our primary objective was to select *MAPT* polymorphisms previously suggested to contribute to the risk of developing idiopathic PD in Chinese subjects. Therefore, we focused on the H1/H2 insertion/deletion polymorphism and rs242562 [11]. PCR products were generated with 50 ng DNA template in 12.5 μl 1 × taq PCR Master MIX (Bioteke Corporation, Beijing, China), 1 μl of 10 μmol/l each primer (Invitrogen, Carlsbad, CA, USA) and 12 μl Ultra-distilled water in a total volume of 25 μl. The PCR conditions used for the H1/H2 insertion/deletion polymorphism were: the H1/H2 insertion/deletion polymorphism forward primer GGAAGACGTTCTCACTGATCTG, reverse primer AGGAGTCTGGCTTCAGTCTCTC, initial denaturation at 94°C for 5 minutes followed by 33 cycles of 94°C for 30 seconds, 59°C for 30 seconds, 72°C for 30 seconds, and a final extension at 72°C for 10 minutes. The PCR conditions used for the rs242562 polymorphism were: rs242562 forward primer: CAGCCTTCCCTGTCCTTGATTC, rs242562 reverse primer; GCCTTCCCAACAGAGCAACC, initial denaturation at 94°C for 5 minutes, followed by 35 cycles of 94°C for 30 seconds, 58°C for 30 seconds and 72°C for 30 seconds, and a final extension at 72°C for 10 minutes. Digestion with *Xho*I restriction enzyme (New England Biolabs, Ipswich, MA, USA) at 37°C yielded a 385 bp band for the G-allele and a 287 bp and a 98 bp for the A-allele. The PCR conditions used for the rs334558 polymorphism were: rs334558 forward primer: GACGTCCGTGATTGGCTC, reverse primer: AGCCCAGAGCCCTGTCAG, initial denaturation at 94°C for 5 minutes, followed by 33 cycles of 94°C for 30 seconds, 62°C for 30s and 72°C for 15 seconds, with a final extension at 72°C for 10 minutes [12]. Digestion with *AluI* restriction enzyme (New England Biolabs) at 37°C yields a 344 bp band for the C allele and 220 and 124 bp for the T allele. Digestion products were resolved on a 2% agarose gel, stained in ethidium bromide solution and visualized with an ultraviolet light.

### Statistical analysis

All statistical analysis was performed by SAS 9.2 (SAS Institute, Cary, NC). A log-odds ratio (OR) and its 95% confidence interval (CI) were estimated with logistic regression for allele wise, genotype-wise recessive/dominant genetic model of each polymorphism. The gene-gene interaction test was conducted by running two models: one with and the other without the interaction term (A-carrier * T-carrier). In addition, a trend test in logistic regression for allelic model was further employed for the effect of *MAPT* rs242562 and *GSK3B* rs334558 on risk for PD [13]. A probability (*P*) of less than 0.05 is considered statistically significant and *P*-values are two-tailed. Adjustment of *P* values for multiple testing uses Bonferroni correction. The power was calculated by Power V3.0 software (http://dceg.cancer.gov/tools/design/POWER) with the observed odds rates and minor allele frequencies for both the 2-df overall association test and the trend test [14].

## Results

**Figure 1:**
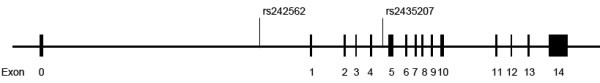
**Schematic of the *MAPT *region and associated polymorphisms.** Previous studies have identified a ~970 kb inversion polymorphism at chromosome 17q21.31, a region that contains *MAPT* and several other genes. Chromosomes with the inverted segment in different orientations represent two highly divergent *MAPT* haplotypes, H1 (direct orientation) and H2 (inverted orientation). No recombination has been identified between these two haplotypes over a region of ~1.5 Mb, but they have accumulated sequence variation independently. Polymorphisms including rs242562 and rs2435207 spanning *MAPT* exons 1 to 4 are specific to the H1 haplotype. Besides, there is a characteristic 238-bp deletion in the *MAPT* intron 9 of the H2 haplotype.

The allele and genotype distribution of *MAPT* rs242562 and *GSK3B* rs334558 in PD patients and controls were summarized in Table 1. The schematic of the *MAPT* region and associated polymorphisms including rs242562 and rs2435207 were shown in Figure 1. Genotype distributions of rs242562 and rs334558 followed Hardy-Weinberg equilibrium for PD patients and controls, no deviations from equilibrium were observed. All the PD patients and controls were of H1/H1 diplotype in our study.

**Table 1:** **Allele and Genotype frequencies of *MAPT *** **rs242562 and *GSK3B *** **rs334558 in PD cases and controls**

	**Case(211)**						**Control(279)**					
	**Number**	**%**	**Number**	**%**	**Number**	**%**	**Number**	**%**	**Number**	**%**	**Number**	**%**
rs242562												
Allele	A		G				A		G			
Total	230	55.02	188	44.98			225	48.91	235	51.09		
Male	149	54.78	123	45.22			140	48.61	148	51.39		
Female	81	55.48	65	44.52			85	49.42	87	50.58		
Age < =50	80	54.05	68	45.95			102	43.97	130	56.03		
Age > 50	150	55.56	120	44.44			123	53.95	105	46.05		
Genotype	AA		AG		GG		AA		AG		GG	
Total	57	27.27	116	55.5	36	17.22	59	25.65	107	46.52	64	27.83
Male	37	27.21	75	55.15	24	17.65	36	25	68	47.22	40	27.78
Female	20	27.4	41	56.16	12	16.44	23	26.74	39	45.35	24	27.91
Age < =50	20	27.23	40	54.05	14	18.92	27	23.28	48	41.38	41	35.34
Age > 50	37	27.41	76	56.3	22	16.3	32	28.07	59	51.75	23	20.18
rs334558												
Allele	C		T				C		T			
Total	260	61.61	162	38.39			330	62.98	194	37.02		
Male	170	62.04	104	37.96			218	63.74	124	36.26		
Female	90	60.81	58	39.19			112	61.54	70	38.46		
Age < =50	97	65.54	51	34.46			163	65.73	85	34.27		
Age > 50	163	59.49	111	40.51			167	60.51	109	39.49		
Genotype	CC		CT		TT		CC		CT		TT	
Total	74	35.07	112	53.08	25	11.85	97	37.02	136	51.91	29	11.07
Male	53	38.69	64	46.72	20	14.6	62	36.26	94	54.97	15	8.77
Female	21	28.38	48	64.86	5	6.76	35	38.46	42	46.15	14	15.38
Age < =50	27	36.49	43	58.11	4	5.41	52	41.94	59	47.58	13	10.48
Age > 50	47	34.31	69	50.36	21	15.33	45	32.61	77	55.8	16	11.59

For *MAPT* rs242562, we found positive results for genotype-wise dominant genetic model in involved subjects (Table 2, shown in bold) with a power of 74.5%. Genotype AG + AA is associated with the higher PD risk in overall involved subjects (AG + AA vs. GG, OR = 1.785, *P* = 0.016), compared to genotype GG, with A as the “putative” risk allele (Table 2). These data suggest that AG + AA genotype of rs242562 may confer risk effect on PD risk in central Chinese population. The ORs and 95% CIs for the case-control studies were not significant for allele-wise model and the recessive model in overall involved subjects with a lower power of 0.048-0.266 (Table 2). Additionally, the test for trend in logistic regression for allelic model also found no significant association with a power of 0.143 (Table 3), which is consistent with the results for allelic model in Table 2.

**Table 2:** **Effect of *MAPT *** **rs242562 and *GSK3B *** **rs334558 on risk for PD**

**SNPs**	**OR (95% CI)**	***P***	**Crude OR (95% CI)**	***P***	Adjusted OR (95% CI)*	***P***	**Crude OR (95% CI)**	***P***	Adjusted OR (95% CI)*	***P***
rs242562 (A/G)	Allele-wise A vs. G		AA vs. AG + GG		AA vs. AG + GG		AG + AA vs. GG		AG + AA vs. GG	
	1.278 (0.980-1.666)	0.071	1.087 (0.711 – 1.662)	0.7	1.065 (0.693 – 1.636)	0.775	**1.853 (1.169 – 2.936)**	**0.009**	**1.785 (1.116 – 2.853)**	**0.016**
Power	0.266		0.058		0.048		**0.793**		**0.745**	
rs334558 (T/C)	Allele-wise T vs. C		TT vs. CT + CC		TT vs. CT + CC		CT + TT vs.CC		CT + TT vs. CC	
	1.060 (0.814-1.381)	0.667	1.080 (0.612 – 1.907)	0.79	1.018 (0.573 – 1.808)	0.951	1.088 (0.746 – 1.588)	0.661	1.058 (0.719 – 1.557)	0.774
Power	0.05		0.044		0.029		0.064		0.064	

* All the OR and *P* values were adjusted for age and gender.

**Table 3:** **The allelic-wise analysis with a test for trend for the effect of *MAPT *** **rs242562 and *GSK3B *** **rs334558 on risk for PD**

**Number of minor alleles**	**Cases**	**Controls**	**Crude OR (95% CI)**	**P value**	Adjusted OR (95% CI)*	P* value
rs242562						
0	36	64	1.000		1.000	
1	116	107	1.927(1.186,3.132)	0.008	1.892(1.154,3.102)	0.011
2	56	60	1.659(0.960,2.868)	0.070	1.628(0.931,2.846)	0.087
Test for trend			1.262(0.964,1.651)	0.091	1.250(0.950,1.646)	0.111
rs334558						
0	74	97	1.000		1.000	
1	112	136	1.079(0.729,1.598)	0.702	1.051(0.704,1.570)	0.807
2	25	29	1.130(0.611,2.089)	0.697	1.091(0.583,2.039)	0.785
Test for trend			1.068(0.806,1.415)	0.647	1.047(0.785,1.395)	0.756

For *GSK3B* rs334558, the allele-wise and genotype-wise ORs lack any statistical significance in overall involved subjects with an insufficient power of 0.029-0.064 (Table 2). The test for trend in logistic regression for allelic model with a power of 0.036 (Table 3) also found null association consistent with the allele-wise model in Table 2.

The gene-gene interaction test was conducted by running two models: with and without the interaction term (A-carrier * T-carrier), and likelihood ratio test indicated that interaction term was not statistically significant (*P* = 0.2140). The effect of GG and age on PD risk remained the same between the two models. We then studied the association between rs242562 and PD at different levels of the rs334558 genotype (Additional file 1: Table S1), and the association between rs334558 and PD at different levels of the rs242562 genotype (Additional file 2: Table S2). The results suggest that the GG genotype of rs242562 displays protection against PD in subgroup with T carrier, although the stratified analysis has a smaller sample size and a lower statistic power.

## Discussion

In this study, we investigated the association of *MAPT* rs242562 and *GSK3B* rs334558 with PD in central China. The main findings of our study in central Chinese population include 1) All the PD patients and controls were in H1/H1 diplotype in our study, 2) GG genotype of *MAPT* rs242562 serves protection effect on PD risk in central Chinese population, 3) Genotype of *GSK3B* rs334558 showed no difference between PD patients and controls, and 4) GG genotype of rs242562 may display strong protective effect against PD risk in subgroup with *GSK3B* rs334558 T carrier.

Our data confirmed that the distribution of the *MAPT* H1 haplotype is common in China, which is in agreement with previous findings [15,16]. Accumulating evidence showed that *MAPT* H1 haplotype-carrier group is a susceptible group of PD [16,17]. This case-control study served as an association study based on population with susceptible haplotype H1 in central China. *MAPT* rs242562 regulates protein encoding despite it is in the intron of *MAPT* gene. The frequency of rs242562 GG genotype is higher in controls compared to that in cases, indicating it may play a protective role. These data are different from a previous study which showed that *MAPT* polymorphism rs242562 revealed no significant difference between PD patients and controls in Germany, Serbia and Greece [6,18,19]. The sample size, ethnic groups and environment may contribute to the different results.

*GSK3B* rs334558 is reportedly associated with PD, but the conclusions were inconsistent. A Greek study was the first to show that *GSK3B* rs334558 was related to PD, while CC served a protective effect and TT was overexpressed in PD [18]. Moreover, a study from Australia showed that the frequency of TT and H1/H1 diplotype in PD patients was significantly higher compared to control subjects [10]. However, the study about LOPD patients in India concluded that CC was a risk factor [20]. In the Spanish population, TT genotype and *MAPT* H1/H1 diplotype were associated with a decreased risk for PD [21]. In the present study, we did not find any significant difference in allele-wise and genotype-wise analysis for rs334558 between PD patients and controls. However, GG genotype of rs242562 may serve a strong protective effect on PD risk in subgroup with T carrier, although the stratified analysis has a small sample size and a low statistic power.

Sex may be involved in the association results of *GSK3B* and neurodegenerative disease [22]. In an Alzheimer's disease research, it was revealed that estrogen receptor and GSK3B could change the tau protein phosphorylation, indicating sex involved in pathogenesis of this neurodegenerative disease [22]. However, following sex stratification the sample size was smaller in our study, and whether hormonal or hormonal receptors change the association of *GSK3B* gene with PD needs to be further investigated.

Although *MAPT* rs242562 and *GSK3B* rs334558 are located in two different chromosomes, the encoded proteins interact with each other. Given the findings that H1 haplotype may act synergistically with variants in the *MAPT* and *GSK3B* genes in conferring risk for PD, gene-gene interactions will also be important to consider as they may provide critical insights into mechanisms of disease susceptibility. In this study, lower power was observed for rs334558 analysis. Thus, the null association should attribute to the insufficient power. Since the sample size of the genotype model including indicator variables of the two polymorphisms is considerably smaller, a larger sample size should be needed in order to further assess this association.

## Conclusions

In summary, the *MAPT* rs242562 is associated with PD in central China, and GG genotype of rs242562 may provide protective effect against PD risk in subgroup with *GSK3B* rs334558 T carrier, although our results are limited by the sample size. Since *MAPT* gene and *GSK3B* gene confer genetic risk for PD, exploring how the gene-gene or gene-environment interactions contribute to dopaminergic neurodegeneration should be further considered. Such knowledge about the mechanisms could open up new windows for early diagnostic and therapeutic interventions in this important neurodegenerative disease.

## Consent

Written informed consent was obtained from all patient and control for the association study. A copy of the written consent is available for review by the Series Editor of this journal.

## Abbreviations

MAPT: Microtubule-associated protein tau; PD: Parkinson's disease; LOPD: Late onset of Parkinson's disease; EOPD: Early onset of Parkinson's disease; GSK3B: Glycogen synthase kinase 3β; TJMC & HUST: Tongji medical college, Huazhong University of Science; OR: Odds ratios.

## Competing interests

We confirm that we have read the Journal’s position on issues involved in ethical publication and affirm that this study is consistent with those guidelines. None of the authors have any conflict of interest to disclose.

## Authors’ contributions

LY, JH, DZ, KG, NX, XL, JX, YZ, PW, ZL, JW, TW contributed to the conception and design. LY, DZ, KG, JX, YW, PW, JH, ZZ took care of the PCR studies. LY, JH, DZ, KG, LL, XL, JX, YZ, ZL, DW, JW, NX, TW analyzed and interoperated the data. LY, DZ, XL, LL, YZ, ZL,DW, JW, NX, TW coordinated all the experiments and helped to draft the manuscript. All authors read, revised and approved the final manuscript.

## Supplementary Material

Additional file 1: Table S1 *MAPT* rs242562 A/G and risk of PD, by *GSK3B* rs334558 C/T. Click here for file

Additional file 2: Table S2 *GSK3B* rs334558 C/T and risk of PD, by *MAPT* rs242562 A/G. Click here for file
