# Supplementary material for: MAPT rs242562 and GSK3B rs334558 are associated with Parkinson’s Disease in central China
Source: BMC Neurosci. 2014 Apr 29;15:54. doi: 10.1186/1471-2202-15-54 (PMC4021090; doi:10.1186/1471-2202-15-54)
Supplement: Additional file 1: Table S1 — MAPT rs242562 A/G and risk of PD, by GSK3B rs334558 C/T. [file 1471-2202-15-54-S1.pdf]

Supplemental Table1. *MAPT* rs242562 A/G and risk of PD, by *GSK3B* rs334558 C/T

Variable	AA vs. AG+GG		AA vs. AG+GG		GG vs. AG+AA		GG vs. AG+AA	
	Crude OR (95% CI)	P	Adjusted OR (95% CI)*	P	Crude OR (95% CI)	P	Adjusted OR (95% CI)*	P
Genotype of rs334558								
CC	0.655 (0.316 – 1.360)	0.2566	0.643 (0.304 – 1.360)	0.2484	0.775 (0.363 – 1.657)	0.5114	0.797 (0.365 – 1.742)	0.5696
CT	1.524 (0.853 – 2.726)	0.1550	1.517 (0.845 – 2.723)	0.1623	**0.429 (0.219 – 0.841)**	**0.0138**	**0.420 (0.212 – 0.835)**	**0.0133**
TT	0.714 (0.142 – 3.600)	0.6835	0.509 (0.085 – 3.038)	0.4584	0.311 (0.087 – 1.109)	0.0717	0.327 (0.079 – 1.356)	0.1233
CT+TT	1.421 (0.838 – 2.407)	0.1918	1.384 (0.813 – 2.355)	0.2310	**0.440 (0.245 – 0.788)**	**0.0058**	**0.470 (0.260 – 0.848)**	**0.0122**
