# Supplementary material for: MAPT rs242562 and GSK3B rs334558 are associated with Parkinson’s Disease in central China
Source: BMC Neurosci. 2014 Apr 29;15:54. doi: 10.1186/1471-2202-15-54 (PMC4021090; doi:10.1186/1471-2202-15-54)
Supplement: Additional file 2: Table S2 — GSK3B rs334558 C/T and risk of PD, by MAPT rs242562 A/G. [file 1471-2202-15-54-S2.pdf]

Supplemental Table 2. ***GSK3B*** **rs334558 C/T and risk of PD, by** ***MAPT*** **rs242562 A/G**

Variable	CC vs. CT+TT		CC vs. CT+TT		TT vs. CT+CC		TT vs. CT+CC	
	Crude OR (95% CI)	P	Adjusted OR (95% CI)*	P	Crude OR (95% CI)	P	Adjusted OR (95% CI)*	P
Genotype of rs242562								
GG	1.500 (0.641 – 3.510)	0.3498	1.559 (0.643 – 3.777)	0.3258	0.748 (0.237 – 2.356)	0.6196	0.700 (0.215 – 2.281)	0.5540
AG	0.955 (0.547 – 1.667)	0.8717	0.986 (0.563 – 1.727)	0.9599	1.564 (0.658 – 3.717)	0.3108	1.502 (0.629 – 3.587)	0.3596
AA	0.520 (0.238 – 1.139)	0.1022	0.532 (0.241 – 1.175)	0.1184	0.722 (0.154 – 3.384)	0.6797	0.661 (0.138 – 3.157)	0.6038
AG+AA	0.812 (0.532 – 1.241)	0.3365	0.835 (0.545 – 1.279)	0.4079	1.305 (0.666 – 2.554)	0.4379	1.234 (0.627 – 2.428)	0.5430
